# Supplementary material for: MetaRibo-Seq measures translation in microbiomes
Source: Nat Commun. 2020 Jun 29;11:3268. doi: 10.1038/s41467-020-17081-z (PMC7324362; doi:10.1038/s41467-020-17081-z)
Supplement: Supplementary file 10 — Supplementary Data 7 [file 41467_2020_17081_MOESM10_ESM.zip › File2/Confidence_High_Taxonomy/162092_out.krona.html]

Javascript must be enabled to view this page.

members
magnitude
magnitudeUnassigned
count
unassigned
taxon
rank

162092\_out

4

4
superkingdom
2

phylum
4
1239

186801
class
4

186802

SRS063370\_contig\_number\_contig-100\_17029.17030SRS144183\_contig\_number\_contig-100\_40411.91912
order
2
4

2
family
186806

1730
2
genus


SRS146764\_contig\_number\_contig-100\_38092.38092
1262892
1
species

142586

SRS018984\_contig\_number\_contig-80\_4706.232504
species
1
